# Equivalence, Justice, Injustice – Health and Social Care Decision Making in Relation to Prison Populations

**DOI:** 10.3389/fsoc.2021.649837

**Published:** 2021-07-14

**Authors:** Andrew Shepherd, Tom Hewson, Jake Hard, Russell Green, Jennifer Shaw

**Affiliations:** ^1^ Offender Health Research Network, University of Manchester, Manchester, United Kingdom; ^2^ Royal College of General Practitioners Secure Environments Group, London, United Kingdom; ^3^ Practice Plus Group, London, United Kingdom

**Keywords:** prison, punishment, healthcare, decision making, equivalence

## Abstract

Prisons represent sites of singular healthcare need–characterized by high levels of distress and disorder. In many jurisdictions, practitioners are ethically charged with delivering healthcare that is “*equivalent*” to that available in the wider community. This claim has been much debated–yet the emergence of a global coronavirus pandemic has highlighted the arguments in a particularly stark manner. In the following conceptual analysis, we explore the emergent discourse of the coronavirus and consider its particular significance for prison healthcare decision making and the concept of equivalence. For example, both the coronavirus pandemic and practice of prison incarceration induce a sense of varied temporality: The discourse of prison is replete in this area–such as the concept of “hard time.” Alongside this, the discourse in relation to coronavirus has highlighted two competing modes of temporal understanding: The political–where the pandemic is conceptualized as has having a discrete “beginning and end”, and the scientific–where the “new normal” reflects the incorporation of the “novel” coronavirus into the wider ecology. The impact of these disparate understandings on the prison population is complex: “Locking down” prisoners–to safeguard the vulnerable against infection–is relatively simple, yet it has traumatic repercussions with respect to liberty and psychosocial health. Easing lockdown, by contrast, is a difficult endeavor and risks collision between the temporalities of prison–where “hard time” is accentuated by separation from the “real world”–the political and the scientific. Whither then the concept of equivalence in relation to a field that is definitively non-equivalent? How can practitioners and policy makers maintain a just ethical stance in relation to the allocation of resources when it comes to a politically marginalized yet manifestly vulnerable population? We argue that further debate and consideration are required in this field–and propose a framework for such discussion.

## Introduction

Prisons, as institutions, can be seen as fulfilling a range of functions in relation to wider society: Ostensibly, in England and Wales, their function is defined as being aligned with the stated goals of the Ministry of Justice, actualized through the agency–Her Majesty’s Prison and Probation Service (https://www.gov.uk/government/organisations/her-majestys-prison-and-probation-service). These goals can be summarized in the “*four Rs of justice*”: Restriction, Retribution, Rehabilitation and Restoration. Restriction–that is the prevention of further harm–and Retribution–punishment–seem self-evident and immediately apparent. Rehabilitation is generally seen as the capacity of organizations to offer training and education to prisoners during the course of their sentence. Restoration–restorative justice (Van Ness and Strong, 2014)–represents a more recent development and represents a duty for the provision of opportunity for offenders to “*make good*” in relation to their victim and host community (Maruna, 2001).

From a healthcare perspective, internationally prisons represent a vulnerable and marginalized population with high rates of morbidity and mortality (Fazel and Baillargeon, 2011): Inequality, altered illness and help seeking behaviors, and prison environmental influences can each be seen as contributing to this outcome. Internationally therefore, prison populations present a challenge to healthcare decision makers in relation to resource allocation and care provision. In the following conceptual analysis, we attempt to explore this field of decision making–taking the prison population of England and Wales and the emergence of the 2020 global novel coronavirus pandemic as a point of illustration. We approach this analysis from the perspective of healthcare researchers and practitioners working in and alongside prisons. We begin this discussion with a psychosocial phenomenological exploration (that is an account of an individual’s lived experience that is grounded in their psychological and social experience of body, mind and culture) of the experience of incarceration, and the impact of the covid-19 pandemic, before addressing the ethical principles that underly the decision-making process in relation to prison health delivery. We argue that this decision-making process, specifically the allocation of resources in the face of limited reserves, is rendered problematic by the very nature of prison as a site of healthcare provision alongside its broader social functions. These difficulties are brought into stark contrast by the novel coronavirus pandemic.

## Discussion

### Prison Incarceration and Healthcare Provision–A Psychosocial Framework

Of the four principles of prison and probation function in England and Wales restriction and retribution are the most obvious. Restriction manifests in the materiality of the environment, the “physical security”, but also in the interaction of prisoners and prison officers as well as the routines and rules of the institution referred to as “relational” and “procedural security” respectively. Foucault et al. (1975) describes the manner in which the regime ensures the predictability of the prisoner’s behavior rendering them visible in an act of demonstrable state power: In this formulation, the visibility of the material prison environment–with perimeter wall and gatehouse–serves as a warning to the wider population.

Despite the visibility of the prison itself its inner workings are opaque to scrutiny from the outside. In this manner prison can come to represent a form of societal Kleinian unconscious, that is a form of experience that is present and impacts on manifest societal experience but which remains largely inaccessible (c.f. Klein, 1987), within which repressed traumatic elements are held and, hopefully, digested or processed. Traumatic events, such as acts of criminal violence, are moved into this space (prison) in the hope that rehabilitative processes can lead to their resolution and the removal of traumatic elements. Perversely, the introduction of such undigested traumatic elements into a toxic environment (such as a dysfunctional, overcrowded, prison system) is more likely than not to promote indigestion. The emergence of prisoners into the community should herald the completion of this process–although public response commonly indicates a wariness with regard to the likelihood of this digestion being seen as completed to a satisfactory degree (Keene et al., 2018). Contrary to Foucault (ibid) this desire for repression and invisibility may partially account for an increasing move to place prisons outside of city centers with the freeing of expensive land in place of less valuable real estate representing a potentially desirable by-product for a money conscious age.

Healthcare provision can be seen as interacting with each of the four pillars of prison and probation. While uncomfortable as a practice, conflicting with many of the commonly held principles of biomedical ethics (Rauprich and Vollmann, 2011), practitioners are routinely called upon to assess the impact of restriction and retribution on an individual prisoner. For example, practitioners may be required to review the presence of physical injuries following a period of restraint by prison officers. Alternatively they may be asked to “sign off” on the holding an individual separate from the main prison population in a “care and separation unit” (CSU–traditionally referred to as “segregation”). In this way the “algorithm” is satisfied by the clinician’s certifying that there is no apparent reason “not” to so segregate the individual. Engagement with mental health practitioners or “psychologically informed planned environments” within the prison network may be seen as evidence of an individual’s engagement with the rehabilitation and parole process. Practitioners may be called upon by parole boards to prepare reports relating to this engagement and may so inform the potential for an individual’s release from custody on license to the wider community. This raises complications with respect to the concept of consent to engage in a therapeutic process. The individual is placed in a double-bind whereby their compliance is forced less they risk further curtailment of their liberty. Psychologically, from a rehabilitation and restoration perspective, maintaining the presence of a victim “in mind” is challenging for the offender, the prison and wider society. Mental health practitioners may also serve a function here wherein therapy can be seen as meeting not only the needs of the individual patient, but wider society more generally–with a victim always occupying the position of a psychodynamic third party in the therapeutic dyad.

### Prison and the Impact of the Novel Coronavirus Pandemic

As in all aspects of life in 2020 the impact of the novel coronavirus within prisons has been considerable. Prisoners are recognized as a vulnerable population disadvantaged both at prison intake owing to social inequality, while within the prison owing to cramped accommodation conditions, and in terms of healthcare access. Prisons have been noted internationally as requiring particular attention and planning in the face of any pandemic (Beaudry et al., 2020). For England and Wales, in an effort to curb transmission rates, prisons have been “locked down” with restrictions enforced limiting visiting rights (reducing direct contact with family and friends), and reducing the amount of time available for association with peers, reaching a maximum of 1 h each day in most prisons. As the pandemic response has become normalized prisons have moved from a position of blanket restriction to a more flexible approach wherein individual prison wings can be locked down in the case of covid-19 infection. Prisoners can be required to self-isolate and failure to do so can result in adjudication, including restraint and transfer to CSU for a period of enforced quarantine. This potentially represents a novel situation for prisoners and one which is unlikely to be replicated more widely for other citizens. The impact of the coronavirus pandemic in England and Wales has been considered in a recent thematic review published by Her Majesty’s Inspectorate of Prisons (Her Majesty’s Inspectorate of Prisons, 2021). The decision to so restrict an individual’s liberty–beyond the fines imposed in the wider population–raises a series of ethical questions including the potential impact on an individual’s mental health through being so restricted, the appropriateness of restriction and restraint in this situation, and the role of punishment for breaching restrictions in prison (prison mutiny) in comparison with the wider community: It is unclear to what extent such considerations are taken into account. While some social visits have been restored, including virtual “purple visits”, many prisoners remained unable to see their families owing to national level restrictions beyond the gradual easing of restrictions seen in the wider community. Locking down the prison population safeguarding the vulnerable therefore proved relatively simple and appears to have had beneficial effects in terms of limiting the spread of infection. Coming out from lockdown is liable to prove more complicated–and the psychological effects of lockdown will likely have a lasting impact (Hewson et al., 2020b). Indeed, a recent report published by the Scientific Advisory Group for Emergencies, United Kingdom (Scientific Advisory Group for Emergencies, 2021) highlights the risk of prisons remaining potential seats of outbreak and infection beyond the lifting of restrictions in the wider community. Prisoners fear a resurgence of violence (Her Majesty’s Inspectorate of Prisons, 2021), that had been temporarily reduced through the mandatory lockdown, and as a result the situation remains uncertain.

In the three following sections of the discussion an overview is provided with respect to particular aspects of prison life that separate prison’s as institutions from the wider community. These illustrations are presented to illustrate the challenges that emerge in terms of healthcare provision and resource allocation in these environments.

#### Incarceration and the Prisoner’s Body in the Context of a Global Pandemic

The body, as physical manifestation of ego or soul, represents one site of suffering and discipline for prisoners. The prisoner’s body is subjected to the regime of the prison itself contained within the physical security of the environment. In this sense the regime of the prison is punctuated by variations in physical experience with the clamor and noise of a busy wing routine being present throughout the working day, shifting to a more subdued yet still noisy night time environment. In this sense an embodied routine is established with the shifting of the individual through the environment.

An enforced lockdown has led to lower rates of covid-19 infection than were originally anticipated (statistical data available through HMPPS https://www.gov.uk/government/statistics/hmpps-covid-19-statistics-december-2020) and an altered profile of emerging infection. The lockdown has also impacted on the sense of routine within prison institutions with prisoners being required to spend as much as 23 h each day behind their cell door. The body is therefore further restricted with access to time out of the cell, or in the fresh air being greatly restricted. This too has been the case for wider populations, with national lockdowns having represented a mainstay in the global pandemic response, however the imposition of physical security exacerbates this situation for prisoners further disempowering an already disempowered population. The recent HMIP report (2021) illustrates this point with prisoners speaking about the impact of isolation and the disruption of access to healthcare provision or other necessary supports for daily living. The physical experience of this restriction is further emphasized by the forced close cohabitation of prisoners in shared cells raising the risk of viral infection and providing little opportunity for dignity as essential hygiene activities could no longer be scheduled for a time when the other prisoner was not in the cell.

Ethnicity becomes a further factor here with Black and Minority Ethnic (BAME) populations being seen as at particular risk in terms of infection and mortality (Patel et al., 2020). In many settings, for example in hospitals, BAME populations have been shielded from frontline work to address this difficulty. In prison however BAME individuals are often already discriminated against in the context of the criminal justice system (Shepherd, 2017) a factor further exacerbated in the pandemic lockdown as illustrated by one example in the HMIP report (ibid) of a prisoner describing seeing advice on television relating to the need for BAME individuals to shield while witnessing a procession of several different prisoners through his own cell as cellmates came and went.

#### Incarceration and the Experience of Varying Temporalities in the Global Pandemic

Prisons, famously, are associated with variations in the experience of time passage–with loss of time generally seen as representing the enactment of “punishment” on the individual. Language is rich with metaphor in this area–with the act of imprisonment described as “doing time” and “hard time”–Cohen and Taylor (1972) observed that the experience of varying temporality during incarceration led to a sense of the world turning at different rates inside and outside of prison. Covid-19 too has introduced a varying sense of time between the scientific sense of a novel agent being incorporated into an ecological system (“living with” or “new normal”) and the political sense that there will be a point at which we “return to normal” (Balzam and Lupo, 2020): This clash of understanding between incompatible end points has manifested in varying interpretations of the manner in which pandemic political policy has been truly “led by the science.”

While, ultimately, the point at which we return to normal does represent a political rather than scientific decision and as such is subjective and open to interpretation. What is introduced is a sense of uncertainty as politicians and the public wrestle with their understanding of the developing situation. This situation is compounded in prison where a disconnection and varied temporality from the wider population manifests. A division between prison time and the wider community will be accompanied by anxiety and distress for prisoners who may struggle to maintain a sense of coherence and purpose in the face of a disrupted routine. This observation is perhaps best exemplified through considering the time that a prisoner spends “on remand” that is awaiting trial and sentencing. Most jurisdictions work to define the time that an individual should spend on remand according to the nature of the alleged crime. However, with the emergence of new ways of working and technological approaches criminal courts have become delayed with a backlog of cases developing and an emergent uncertainty for unsentenced prisoners. This period of remand is well recognized as representing a particular psychological stressor–associated with higher rates of deliberate self-harm and suicidality (Marzano et al., 2011).

Regime and routine represent important means of marking the passage of time within prison but as has been noted such routines have been further disrupted by the pandemic. The loss of predictability risks a series of unanticipated effects within the environment. For example, while the HMIP thematic review (2021) notes a reduction in violence some prisoners describe a fear of a building pressure, contained by lockdown, that may not remain forever contained.

Left in their cells prisoners are forced to seek distraction to pass time. Television occupies a particular role within prison in terms of cultural significance (Knight, 2015) but television in England and Wales represents a privileged item that is not available universally. Distraction packs are provided to prisoners, but these assume a degree of literacy and as such many are left unable to engage with such activity (Her Majesty’s Inspectorate of Prisons, 2021) and resort to substance use as a way of avoiding boredom.

Additionally, the impact of covid-19 on the experience of individuals during their prison sentence has been recognized within sentencing guidelines–and is generally seen as leading to a reduction in the length of sentence to be served (Sentencing Council guidance, 2020). Predictability, in terms of routine, can be seen as part of the prison regime–producing discipline and punishment as argued by Foucault (ibid)–but also predictability for prisoners who may have experienced extreme unpredictability in the past: Anecdotally, many prisoners report the benefits of such predictability in adding to their engagement and capacity to work with clinicians in the prison environment.

In summary, prison time has long been recognized as varying in terms of the experience of prisoners vs. the passage of time in the wider community. Covid-19 represents a further disruption to this process with an additional complication of the prison routine leading to likely distress for prisoners who have little power to directly resist such change or to develop new routines through which to better manage their experience.

#### Community Cohesion and Isolation

Social response to the imposed restrictions has been variable–from a seeming wide-spread acceptance through to active protest against the restriction. The risk of infection has introduced an imperative that we remain socially distant in a manner not previously observed–we foreswear touch, embrace and mask our faces in a manner not previously observed in many cities and towns around the World. In this manner we have in many cases become atomized–separated even from families with fear that winter isolation and loneliness may reach higher levels.

Yet, the pandemic has also witnessed moments of solidarity–such as the communal clap for our carers on a weekly basis during the height of an initial lockdown in the United Kingdom (an initially spontaneous then later organized sequence of events in which the public took to the streets outside their homes at a set time each week to display their gratitude for the efforts of frontline workers). Other examples have also emerged–such as the sharing of music and concerts from balconies in city centers and residential areas. Early studies suggest a general decline in crime rates during lockdown (Boman and Gallupe, 2020). Solidarity has not been a universal response however as illustrated by the rise in rates of domestic violence (Bullinger et al., 2020). What drives the variation in crime is unclear; whether it is a representation of solidarity or communality, or alternatively simply a reduced level of opportunity.

As noted above, prisons are radically separated from their host communities–both psychologically and physically, and this separation has in many ways been exacerbated during the course of the pandemic. Initial data suggested that rates of deliberate self-harm (understood in this context as a manifestation of mental distress) may have decreased during lockdown (Hewson et al., 2020a)–although the overall impact is yet to be determined and the lengthening of time on remand, and increased loneliness (Brown and Day, 2008), are liable to still have a significant effect.

Prisons represent communities, not necessarily completely isolated in total institutions (Goffman, 1961; Schliehe, 2016), but informed by host communities in an intimate fashion (Irwin and Cressey, 1962). As communities continue to adapt to changes in restriction and risk of infection, both in prison and the wider community, the impact on the population as a whole and prisons in particular must be born in mind to ensure that the prison population is not further marginalized and left behind.

### Resource Allocation and Decision Making in Relation to Prison Healthcare

Prisons therefore represent particular sites of suffering and marginalization requiring particular attention and the investment of care resources. For example in the development of a vaccine rollout strategy (Siva, 2020). However, as the crisis of the pandemic has evolved prisons have often been overlooked (Her Majesty’s Inspectorate of Prisons, 2021 and Scientific Advisory Group for Emergencies, 2021). The question arises as to why this is the case and, if that outcome is deemed unacceptable, what steps can be taken to address this?

One commonly applied argument to resource allocation for prison–in relation to the wider community–is that of equivalence. Interpretation of the meaning of equivalence has varied however between equivalence of input and, the more desirable, equivalence of outcome (Charles and Draper, 2012, or Royal College of General Practitioners position statement-https://www.rcgp.org.uk/about-us/news/2018/july/prison-health-is-public-health.aspx). For example, it could be argued that a prison within a local community should attract resources in keeping with the size of its population and health and social care need: This would represent an equivalence of input and given the significant burden of disease in the environment it is likely the amount of dedicated resource would be greater than for a similar population when compared, for example, in terms of age. Equivalence of outcome by contrast would recognize that the prison population is disadvantaged even before incarceration and risks remaining disadvantaged on release: Equivalence of outcome would therefore demand a broader scope and focus on the experience of individual’s through the prison pathway and beyond. This would likely represent a still greater resource investment. Given the unequal field in terms of starting point and resources (for example in the case of digital resources–Edge et al., 2020) as well as the challenges of working in a prison environment (Birmingham et al., 2006) the application of such a principle may represent a challenge. It is unclear whether there is a political desire, or public will, to address this issue.

Already disparate and unequal in terms of resource access in comparison to the wider population prison populations, locked down as they are, risk falling further behind. An opportunity presents itself in the form of technological innovation, such as telemedicine (Edge et al. ibid), to further address this disparity. However, again, the opaque nature of the prison environment as well as stigmatizing attitudes toward offender populations (West et al., 2014) raises the risk of this opportunity being missed further impacting on the significant burden already posed by the pandemic.

The need to address this challenge is explicitly laid out in the UN Declaration on the Basic Principles for the Treatment of Prisoners (United Nations, 1990). The question remains however, how should the principle of equivalence be upheld in a time of scarce resources and fast moving novel demands on healthcare provision? Bald application of a utilitarian position risks an assumption that all variables can be known and that unknown unknowns will not impact on any necessary calculation. In the face of a novel healthcare crisis, where the impact of delay or action are difficult to weigh, such an approach may be inappropriate–and we must fall back on a basic principle of preserving the dignity and rights of human beings. Holding this basic position of principles and rights in mind is complicated however. As indicated above, prison comes to represent something of a psychosocial blind spot for wider society where the suffering contained within the walls is rendered invisible, arguably deliberately so. Rather than an unconscious repression this may in fact represent a conscious act of suppression of trauma. How then are policy makers to avoid this act of heuristic bias or to convince the wider public of a “community dividend” from the just treatment of prisoners?

Psychotherapeutic practice has something to say here–with respect to the need of the forensic therapist (psychotherapist working with offender populations) to be able to hold in mind the trauma not only of the individual they are working with in the therapeutic frame but also the wider implications of the individual’s trauma and acts of violence against an individual victim or society more generally. At the group level such trauma manifests in a desire to close ranks, to identify with those similar to us and to avoid novel confrontations with the other. Divisive partisan election campaigns and collective responses to acts of repressive brutality have not ceased during the coronavirus pandemic–which has seen new meaning imbued in the words “I can’t breathe”, highlighting once more the experience of BAME communities at this time. A necessary duty is placed on policymakers, considering the division of healthcare resources, to confront aspects of societal trauma and hold in mind the experience of individuals in prison as well as in wider society. Such an act is challenging, drawing with it a sadistic urge to punish those who disobey rules and expectations before looking away. Instead a return to the fact that it is the loss of time that acts as punishment in prison, not the environment or support accessed therein is required. This leads to a radical position that society’s wider aims may be better addressed by taking account of the needs of prisoners, that to embrace the shadow of the social ego (social identity, community coherence) is a necessary step in a desirable process of integration.

## Conclusion

Globally, prisons represent particular sites of distress and suffering–which echo the wider ramifications of crime and punishment for society. Maintaining attention on this suffering, and allowing its full digestion is challenging for therapists, society and policy makers. Yet, maintaining attention is necessary in order to avoid a process whereby, through turning away, the healthcare needs of prisoners are overlooked, or simply ignored. It is argued that this tendency to overlook is an implicit psychological process, a desire to not look directly at trauma (and death) in order to spare ego anxiety and distress. This is understandable, yet in the face of a global pandemic may be inexcusable and risks a situation wherein the “system” is addressed rather than any individual, or group, experience, a debate that mirrors the wider international discourse for the addressing of competing priorities in the scope of a covid-temporality. Justice is often portrayed as a blind avatar yet this may be inappropriate: While equality and equivalence are to be embraced it is equivalence of outcome that must carry the day and drive decision making in this field. The impact of the coronavirus pandemic represents a global tragedy the lasting impact of which is yet to be fully appreciated. It is essential that the tendency to overlook prisons as sites of suffering does not lead to opportunities being squandered or the emergence of further inequality. Equivalence of outcome will require a firm focus of attention on the experience of prisoners to ensure that lessons can be learned appropriately for the future.
